# Supplementary figures and images for: Identification of coexisting Mfrprd6 and Pde6brd10 mutations causing spontaneous retinal detachment in commercially available rd6 mice
Source: PLoS One. 2025 Sep 23;20(9):e0332446. doi: 10.1371/journal.pone.0332446 (PMC12456819; doi:10.1371/journal.pone.0332446)

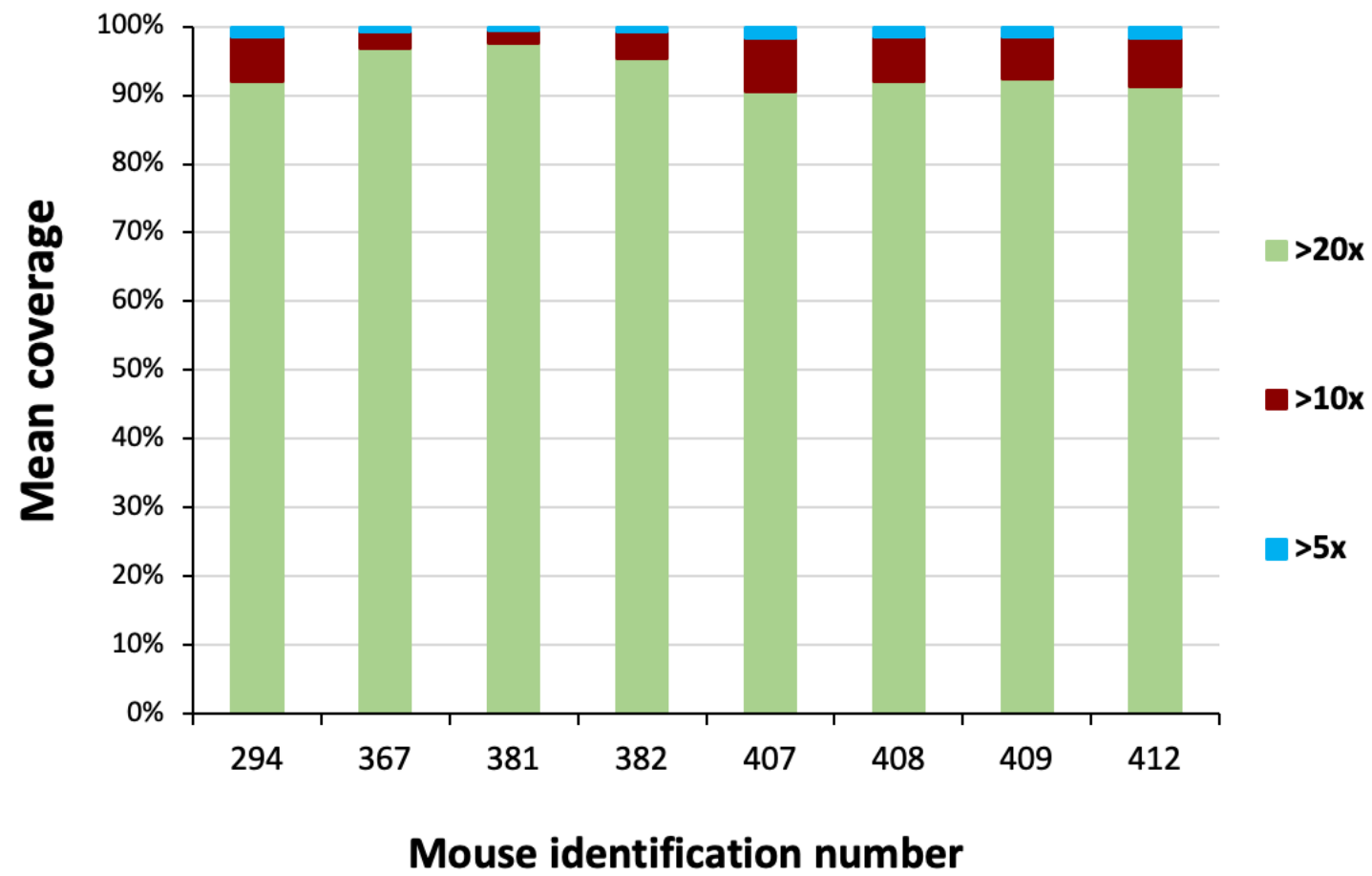

Supplement: S1 Fig — Genomic fractions analyzed by the indicated coverage are shown by colors. (PDF) [file pone.0332446.s001.pdf]

# C57BL/6J

4 W

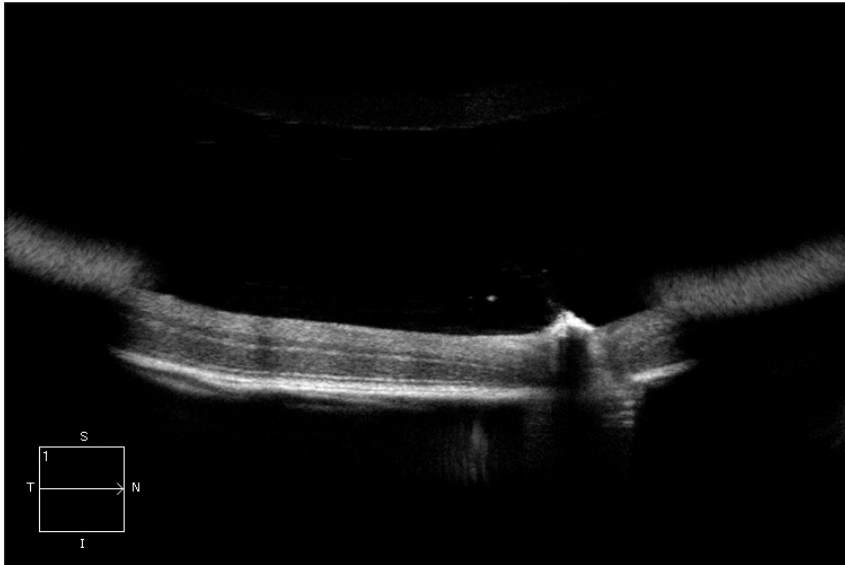

6 W

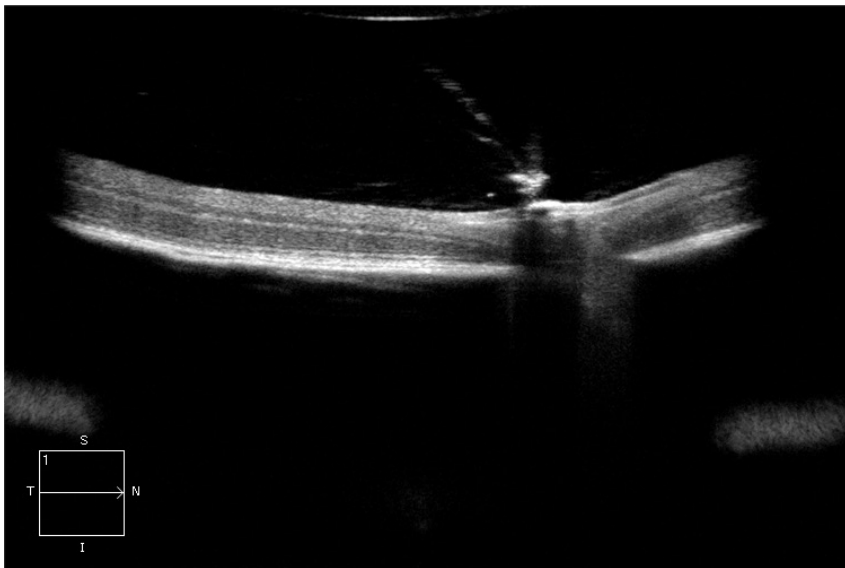

11 W

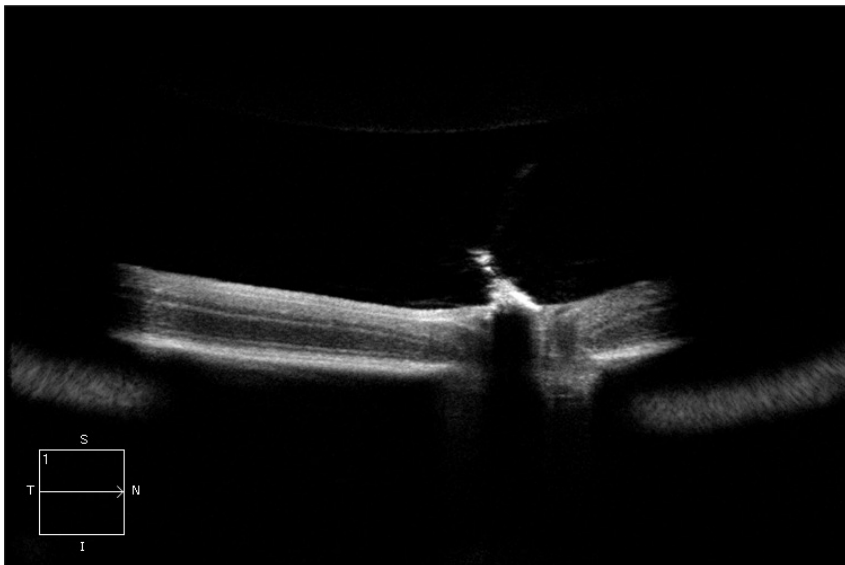

rd6

4 W

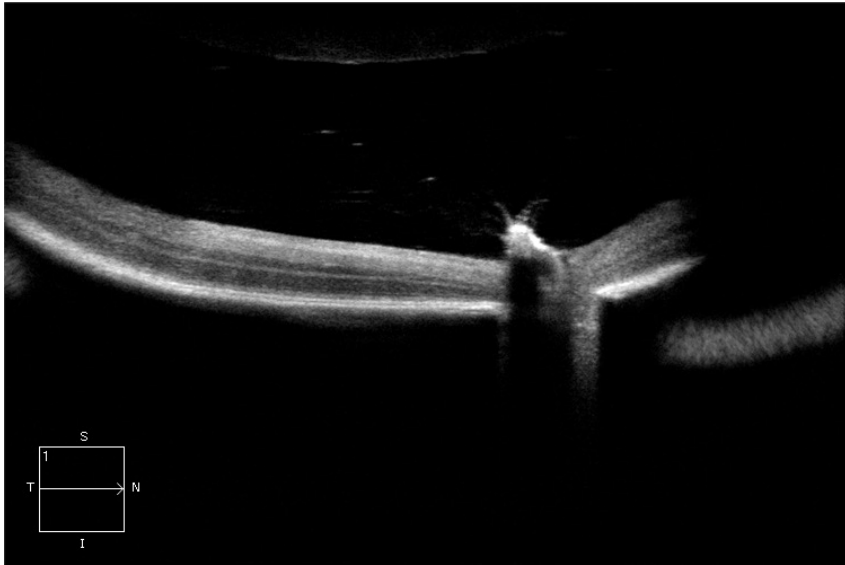

6 W

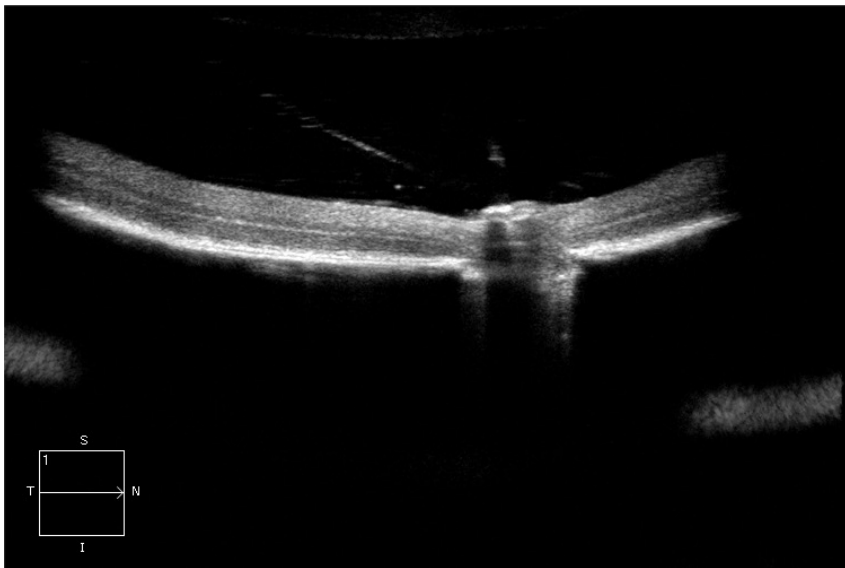

11 W

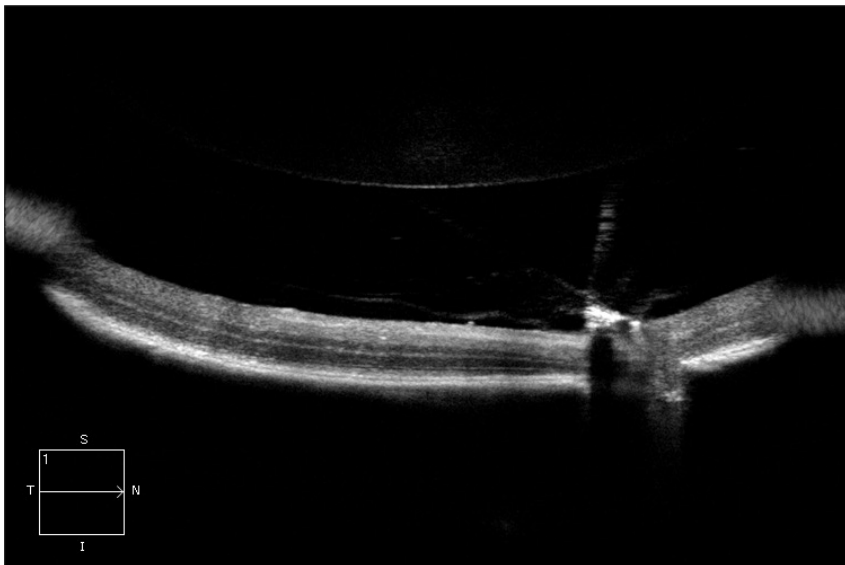

# rd6-RD

4 W

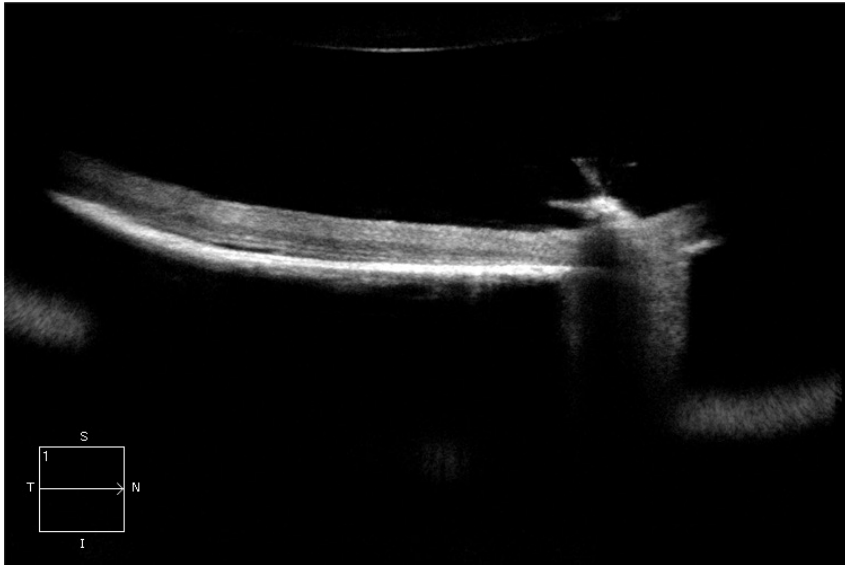

6 W

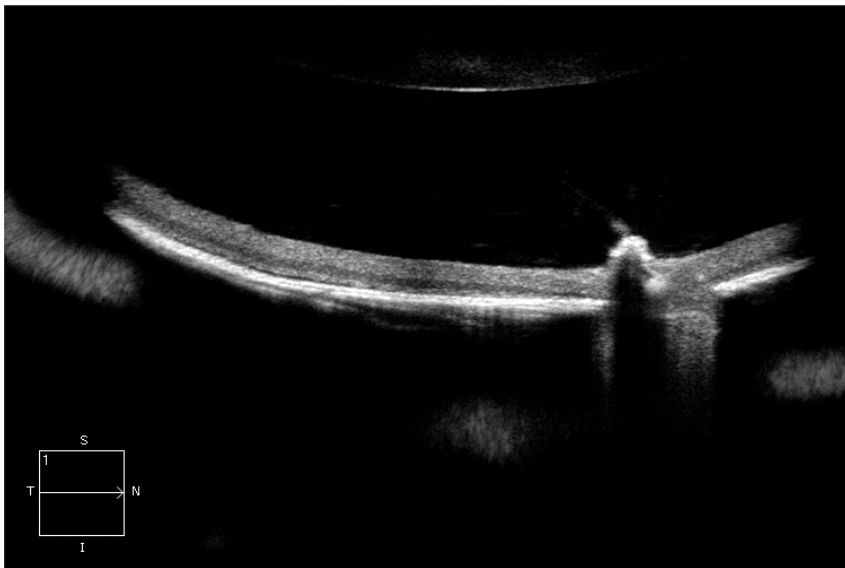

11 W

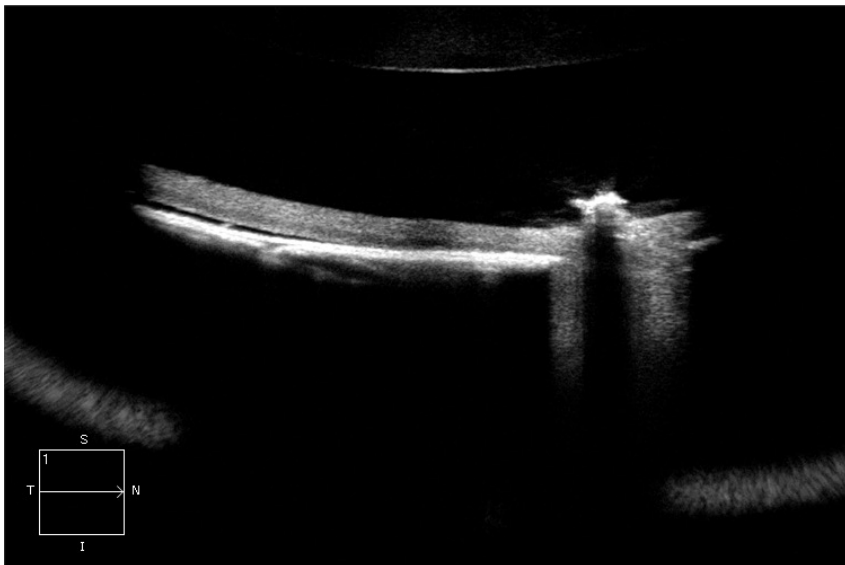

Supplement: S2 Fig — (PDF) [file pone.0332446.s002.pdf]
