# Supplementary material for: Identification of coexisting Mfrprd6 and Pde6brd10 mutations causing spontaneous retinal detachment in commercially available rd6 mice
Source: PLoS One. 2025 Sep 23;20(9):e0332446. doi: 10.1371/journal.pone.0332446 (PMC12456819; doi:10.1371/journal.pone.0332446)
Supplement: S1 Table — (PDF) [file pone.0332446.s003.pdf]

S1 Table

Prkcq_ex1_F	AAGCGGCCGCAGCTAAGCAAGAAGAGACTGCT
Prkcq_ex2_F	AAGCGGCCGCTGTTTGCCTTGCTGTGCAG
Prkcq_ex3_F	AAGCGGCCGCACTCTCAGAGTGAAGCAAAGCA
Prkcq_ex4_F	AAGCGGCCGCGAAGGAATGAATTGTCAAGGCA
Prkcq_ex5_F	AAGCGGCCGCGTCACACCACTGTCACATTGC
Prkcq_ex6and7_F	AAGCGGCCGCCTTGGCAAGGGGTACGTGT
Prkcq_ex8_F	AAGCGGCCGCTCCTCCTCCTGACATGGTCA
Prkcq_ex9_F	AAGCGGCCGCTCTGGGCCTCTTTCCTCCT
Prkcq_ex10_F	AAGCGGCCGCAGAGAAAGAGAGAGAGAGAGAAAGA
Prkcq_ex11_F	AAGCGGCCGCTGCCTCCTCAGTTCTGGGA
Prkcq_ex12_F	AAGCGGCCGCGCCAGCCTGGTCTACAAAGT
Prkcq_ex13_F	AAGCGGCCGCGCAGACACCACCCTATGCA
Prkcq_ex14_F	AAGCGGCCGCCACTGGAATTAAGCCAAAGTGGG
Prkcq_ex15_F	AAGCGGCCGCTGACAGAACCCCTAAGTTGCA
Prkcq_ex16_F	AAGCGGCCGCGTCATTGCACTCAACGCCA
Prkcq_ex17_F	AAGCGGCCGCGCAGCATACGCCAAAGGT
Prkcq_ex18_1_F	AAGCGGCCGCTGCAGAACATTCCCAAGTTGA
Prkcq_ex18_2_F	AAGCGGCCGCAAGAAACACTGCAAAGGCAGG
Prkcq_ex18_3_F	AAGCGGCCGCTCCCAAGGCAGAGGAAAGTAAAGG
Prkcq_ex1_R	AAGCGGCCGCAAGGTCGCCAGCACACAA
Prkcq_ex2_R	AAGCGGCCGCTGGCCTTTGTTGGTCACC
Prkcq_ex3_R	AAGCGGCCGCAGCTGTGTGTGGTAGCACA
Prkcq_ex4_R	AAGCGGCCGCCACTTACACTGTACTCATGTGTTTG
Prkcq_ex5_R	AAGCGGCCGCAGCCCACTGACTGCCATTC
Prkcq_ex6and7_R	AAGCGGCCGCGTGCCCCCTTGAACTCACA
Prkcq_ex8_R	AAGCGGCCGCGACTGCTTAGTGAGGTGGCA
Prkcq_ex9_R	AAGCGGCCGCCTCAAAGGTGGGATTATGGGGA
Prkcq_ex10_R	AAGCGGCCGCAAATAGGGGCCTTGAGGTTG
Prkcq_ex11_R	AAGCGGCCGCGGGGCCAAAGATCCATAGGG
Prkcq_ex12_R	AAGCGGCCGCTGCCGGTCCCGTGTTTTT
Prkcq_ex13_R	AAGCGGCCGCGGGACCTGACACCTTCTCCT
Prkcq_ex14_R	AAGCGGCCGCAGGAGGAGCGGACAAGTCA
Prkcq_ex15_R	AAGCGGCCGCACTCAAAAGACCCATTTGTGTGTC
Prkcq_ex16_R	AAGCGGCCGCGCCATCTCTGCAGCCTTGT
Prkcq_ex17_R	AAGCGGCCGCAGCAACAGCAACACCCCT
Prkcq_ex18_1_R	AAGCGGCCGCACAAGGAGCTCAGCAGTCC
Prkcq_ex18_2_R	AAGCGGCCGCCCTTCCCTGTTGGCGACAAA
Prkcq_ex18_3_R	AAGCGGCCGCGATGGACCTATGTCTTCGAGAA
