# Supplementary material for: Identification of coexisting Mfrprd6 and Pde6brd10 mutations causing spontaneous retinal detachment in commercially available rd6 mice
Source: PLoS One. 2025 Sep 23;20(9):e0332446. doi: 10.1371/journal.pone.0332446 (PMC12456819; doi:10.1371/journal.pone.0332446)
Supplement: S1 File — (DOCX) [file pone.0332446.s006.docx]

**S1 Protocol**

**Genotyping**

Mouse ear DNA was isolated with QIAGEN DNeasy Blood & Tissue Kit according to the manufacturer’s protocol (QIAGEN, Venlo, Netherlands).

*Mfrp^rd6^* and *Pde6b^rd10^* mutation sites was amplified by PCR using the following primer sets: rd6-F- ACCAAGAACCCACTGCTCAC, rd6-R- GGAGAGAGGTCTTCCCCAAC; rd10-F- CTTTCTATTCTCTGTCAGCAAAGC, rd10-R- CTTTCTATTCTCTGTCAGCAAAGC. PCR products were purified using Wizard SV Gel and PCR Clean-Up system (Promega) and sequenced by Sanger method at Eurofins Genomics K.K. (Tokyo, Japan)

**Deep sequencing**

All 18 exons of Prkcq were amplified by PCR using primers containing a NotI linker (supplementary table 1). The PCR products were pooled followed by purification using QIAquick® PCR Purification Kit (QIAGEN). The pooled amplicons were digested with NotI followed by purification. An aliquot of 2.5 μg purified DNA was ligated with T4 DNA ligase at 16℃ for 5 hours. A sequence library was constructed from the ligation product using NEBNext Ultra II DNA Library Prep Kit for Illumina (New England Biolabs). The library was sequenced by Illumina MiSeq with a 150 bp paired-end protocol.

**Whole-genome sequencing**

A sequence library was constructed from 100 ng of genomic DNA using the TruSeq Nano DNA Library Preparation Kit (Illumina, San Diego, CA). The library was sequenced by NovaSeq with a 150 bp paired-end protocol.

Obtained sequencing reads were aligned to the mouse reference genome mm10 using Isaac aligner (version 01.15.02.08) with a base quality cutoff of 15 (Bioinformatics 2013, 29(16), 2041–3). Single nucleotide variants (SNVs) and small insertion and/or deletions (indels) were identified by Isaac Variant Caller (version 2.0.13). SNVs in a tandem repeat region identified by tandem repeats finder (Nucleic Acids Res 1999, 27, 573–80) and indels in RepeatMasker ([http://www.repeatmasker.org/](http://www.repeatmasker.org/)) were removed from the analysis. The variants were annotated by SnpEff (version 4.1).
