# Supplementary material for: Identification of coexisting Mfrprd6 and Pde6brd10 mutations causing spontaneous retinal detachment in commercially available rd6 mice
Source: PLoS One. 2025 Sep 23;20(9):e0332446. doi: 10.1371/journal.pone.0332446 (PMC12456819; doi:10.1371/journal.pone.0332446)
Supplement: Attachment — Submitted filename: Response to Reviewers.doc [file pone.0332446.s007.doc]

Journal Requirements:

1.Please ensure that your manuscript meets PLOS ONE's style requirements, including those for file naming. The PLOS ONE style templates can be found at

https://journals.plos.org/plosone/s/file?id=wjVg/PLOSOne_formatting_sample_main_body.pdf and

https://journals.plos.org/plosone/s/file?id=ba62/PLOSOne_formatting_sample_title_authors_affiliations.pdf

**Response:** According to manuscript body formatting guidelines, we checked and created our manuscript.

2. To comply with PLOS ONE submissions requirements, in your Methods section, please provide additional information regarding the experiments involving animals and ensure you have included details on (1) methods of sacrifice, (2) methods of anesthesia and/or analgesia, and (3) efforts to alleviate suffering.

**Response:** According to the experiments involving animals, we added the sentence. (1) methods of sacrifice; We added “Exsanguination was performed under deep anesthesia induced by ketamine/xylazine as the method of euthanasia.” in Animal husbandry. (2) methods of anesthesia and/or analgesia; We added “Mice were treated with an intraperitoneal administration of a combination of ketamine (Daiichi Sankyo Co., Ltd., Tokyo, Japan) and xylazine (Bayer Medical, Ltd., Tokyo, Japan) at 100 and 10 mg/kg body weight, respectively.” in Animal husbandry. (3) efforts to alleviate suffering; To ensure ethical approval and adherence to guidelines, the approval number from the institutional animal care and use committee and the name of the applicable guidelines are provided in the Animal husbandry section.

3. Please note that funding information should not appear in any section or other areas of your manuscript. We will only publish funding information present in the Funding Statement section of the online submission form. Please remove any funding-related text from the manuscript.

**Response:** All funding-related information has been deleted from the manuscript text.

4. We note that your Data Availability Statement is currently as follows: All relevant data are within the manuscript and in Supporting Information files.

- The values behind the means, standard deviations and other measures reported;

- The values used to build graphs;

**Response:** The values used to generate the graphs in Fig. 3b, Fig. 5c, and Fig. 6c are summarized in a list (S2 Table). We have added a title to S2 Table in the Supporting Information.

- The points extracted from images for analysis.

**Response:** We have prepared a file containing the raw data used for Figure 3a (as well as Figures 1 and 2), which is provided as S2 Fig. A title for S2 Fig has been added in the Supporting Information.

5. Please review your reference list to ensure that it is complete and correct. If you have cited papers that have been retracted, please include the rationale for doing so in the manuscript text, or remove these references and replace them with relevant current references. Any changes to the reference list should be mentioned in the rebuttal letter that accompanies your revised manuscript. If you need to cite a retracted article, indicate the article’s retracted status in the References list and also include a citation and full reference for the retraction notice.

**Response:** We have corrected the reference list and confirmed that it is complete and accurate.

Reviewer #1: This study provides valuable insights, but there are concerns regarding the results.

The results section does not directly mention electroretinography (ERG) or histopathology in rd10 mice, but the discussion section does mention ERG and histopathology in rd10 mice. Based on the results of whole genome sequencing, etc., does this mean that all rd6-RD mice have the Pde6 rd10/rd10 mutation?

**Response:** Thank you for your question. In this study, we did not perform analyses on rd10 mice; therefore, we do not have ERG or histopathological results for rd10 mice. In the Discussion, we referred to rd10 because we identified the rd10 mutation through whole-genome sequencing. The statement means that all rd6-RD mice have the Pde6 rd10/rd10 mutation.

Reviewer #2: When working with spontaneously occurring animal models, it is essential to remain alert to the possibility of additional pathogenic mutations beyond those already reported. This manuscript underscores the previously unrecognized presence of the rd10 mutation in commercially available rd6 mice, delivering an important cautionary message to researchers who rely on this model. Both the experimental design and the overall presentation are clear and persuasive.

My primary concern is that the conclusion—that severe retinal degeneration and retinal detachment in the rd6RD line arise from a combination of rd6 and rd10 mutations—is based solely on short-read whole-genome sequencing (WGS) data from a single mouse family. Because the Pde6b (c.1678C>T, p.R560C) mutation can be readily verified by Sanger sequencing, expanding the analysis to a larger cohort of animals would greatly strengthen the evidence. In addition, since rd10 mice are commercially available, crossing them with rd6 mice lacking the Pde6b mutation would test whether the rd6RD phenotype is truly reproducible, further enhancing the robustness of the conclusions.

**Response:** Thank you for your question. We have prepared S3 Table to address the comment regarding expanding the analysis to a larger cohort of animals. As Supplementary Information, we have included a table summarizing the correlation between phenotype and genotype. While the original submission included 10 animals, we have added additional data to bring the total to 18 animals. Accordingly, we revised the last four sentences of the Results section and added the title for S3 Fig. in the Supporting Information.

As you pointed out, since rd10 mice are commercially available, crossing them with rd6 mice lacking the Pde6b mutation would allow us to test whether the rd6RD phenotype is truly reproducible, thereby further strengthening the robustness of our conclusions. We would like to consider this in our future studies.
