# Supplementary material for: Identification of coexisting Mfrprd6 and Pde6brd10 mutations causing spontaneous retinal detachment in commercially available rd6 mice
Source: PLoS One. 2025 Sep 23;20(9):e0332446. doi: 10.1371/journal.pone.0332446 (PMC12456819; doi:10.1371/journal.pone.0332446)
Supplement: Attachment — Submitted filename: Response_to_Reviewers_auresp_2.doc [file pone.0332446.s008.doc]

Journal Requirements:

In the present study, the authors demonstrated the phenotype-genotype correlation in the rd6-RD mice. The manuscript is well-written, however the authors should address my concerns before publication.

1. It seems that the authors did not mention why the retinal detachment occurred in rd6-RD mice. It would be better to add the molecular biological consideration in the Discussion section.

**Response:** We thank the reviewer for this valuable comment. We agree that a molecular biological consideration regarding the occurrence of retinal detachment in rd6-RD mice should be included. We have now expanded the Discussion section to address this point on Page 16 and 17.

Previous studies have demonstrated that *Mfrp* deficiency impairs the development and maintenance of photoreceptor outer segments via RPE dysfunction [16] and causes RPE atrophy and consequent photoreceptor degeneration [17]. Moreover, recent biochemical analysis revealed that *Mfrp* functions as a molecular hub in the apical membrane of RPE cells, coordinating lipid homeostasis and protein trafficking [18].

Previous reviews have detailed the mechanisms underlying phototransduction failure and photoreceptor cell death in rd10 mice [20] and recent comparative studies have demonstrated that the *rd10* mutation induces protein mislocalization and instability, leading to cGMP-mediated cell death [21]. In this study, rd6-RD mice were found to harbor both the *Mfrp* mutation, which is associated with impaired RPE function and defective photoreceptor outer segment phagocytosis, and the *Pde6b* mutation, which causes rapid photoreceptor degeneration due to impaired phototransduction. The combination of these genetic defects may lead to marked structural instability of the outer retina, disruption of the outer limiting membrane, and weakening of adhesion between the neural retina and the RPE. Moreover, accelerated photoreceptor cell death in rd6-RD mice could promote extracellular matrix remodeling and microglial activation, further compromising retinal integrity. These molecular and cellular alterations may act synergistically to facilitate the development of non-rhegmatogenous retinal detachment in this model.

2. Is there any similarity in gene expression between Mfrp and Pde6b?

**Response:** We appreciate the reviewer’s question regarding potential similarities in the expression profiles of *Mfrp* and *Pde6b*. We added the Discussion section to address this point on Page 17.

According to previous transcriptome analyses and public expression databases [22], both *Mfrp* and *Pde6b* exhibit retina-enriched expression, although they are predominantly localized to different retinal cell types. Mfrp is expressed mainly in the RPE and ciliary body [16], whereas Pde6b is expressed specifically in rod photoreceptors, where it plays an essential role in phototransduction [20]. Despite these differences in cell-type localization, transcriptome studies of retinal degenerative models have reported that loss-of-function mutations in either gene can lead to downstream dysregulation of common pathways, including photoreceptor survival, oxidative stress responses, and inflammation-related signaling [23, 24]. Therefore, while the upstream expression sites differ, there is overlap in the secondary gene expression changes observed in degenerating retina due to Mfrp or Pde6b dysfunction.
